# Neglected Primary Omental Pregnancy after Laparoscopic and Medical Treatment: A Difficult Diagnosis?

**DOI:** 10.1155/2013/207307

**Published:** 2013-06-02

**Authors:** Federica Martelli, Caterina De Carolis, Carmelo Parisi, Emilio Piccione

**Affiliations:** ^1^Department of Biomedicine and Prevention, University of Rome Tor Vergata, Viale Oxford 81, 00133 Rome, Italy; ^2^Department of Obstetrics and Gynecology II, San Giovanni Hospital, Via dell'Amba Aradam 9, 00184 Rome, Italy

## Abstract

The following case report describes a rare case of omental pregnancy in a fertile 34-year-old woman at 5 + 3 weeks of gestation who presented with abdominal pain. Clinical examination, vital signs, and laboratory values were within normal limits, so the woman was hospitalized and monitored. Laparoscopic exploration was performed according to the preoperative diagnosis of tubal pregnancy, but it showed normal pelvic organs. In view of the growth of the *β*-HCG value, a medical approach was attempted, without success. Due to hemodynamic instability, an emergency laparotomy was performed, and it showed an omental pregnancy, confirmed at the pathological examination.

## 1. Introduction

Ectopic pregnancy occurs in about 1%-2% of pregnant woman [1], and its risk is increased approximately 2-fold for infertile women. In recent years, complications due to ectopic pregnancy have been significantly reduced because of the possibility of early diagnosis, medical treatment, and minimally invasive surgery. The present case report illustrates a case of ectopic pregnancy in which both laparoscopic and medical approaches have failed in the management of a primary omental pregnancy.

## 2. Case Presentation

A fertile 34-year-old woman gravida 3 para 2, with two previous vaginal deliveries and no other surgical interventions, was admitted to the gynecological emergency room of our hospital for lower abdominal pain at 5 + 3 weeks gestation. At the clinical examination, vital signs were stable, blood pressure (BP) was 110/80 mmHg, and the abdomen was nontender and treatable. The external genitalia and cervix were normal without vaginal bleeding, the uterus was normal in size and position, and no adnexal masses were appreciated. The patient complained pain in the left site fornix during bimanual examination. Transvaginal ultrasound exam showed a normal uterus with an endometrial tickness of 12 mm, no intrauterine gestational sac, and an hyperechoic mass of 17 × 19 mm in the left ovary. The patient's haemoglobin (Hb) was 12,40 gr/dL, and the level of *β*-subunit of human chorionic gonadotropin (*β*-HCG) was 15620 mIU/mL. The woman was hospitalized, and vital signs were monitorized. After 5 hours, both blood pressure and haemoglobin values decreased (BP 90/60 mmHg, Hb = 11.40 gr/dL). With the presumptive diagnosis of a ruptured ectopic pregnancy, an exploratory laparoscopy was performed (day 0). After aspiration of 200 cc of peritoneal blood, the uterus and both ovaries and fallopian tubes appeared to be normal without any sign of ectopic pregnancy. No obvious bleeding source was found at the exploration of pelvis, omentum, bowels, and puch of Douglas. The day after laparoscopy, abdominal drainage contained 150 cc of blood, Hb was 10.3 gr/dL, BP was 95/60 mmHg, and serum *β*-HCG was 13498 mIU/mL. Transvaginal ultrasound was performed, and it shows minimal amount of fluid in the cul de sac with normal uterus and adnexus. In view of the *β*-HCG value, the patient was counseled and a medical treatment with intramuscular methotrexate injection was started. At day 3, *β*-HCG levels were 14.052 mIU/mL, Hb was 10.10 gr/dL, and BP was 90/60 mmHg. Ultrasound examination was repeated, and it showed normal uterus and adnexa, with a large amount of free fluid in the pelvis. An emergency laparotomy was performed through Pfannenstiel incision, and 1500 cc of peritoneal blood were drained. The uterus and both fallopian tubes were found to be normal. Further exploration of the abdomen revealed a 4 cm lesion covered with a blood clot with active bleeding in the lower edge of omentum, near the transverse colon. The lesion was removed by partial omentectomy. The remaining omentum and other abdominal organs were normal and no fresh bleeding from any other site was identified. Intraoperatively, two units of packed red cells were transfused. At day 5, vital signs were stable, and serum *β*-HCG level sharply decreased to 2421 mUI/mL. The patient was discharged at day 10 and followed up as an outpatient until a total negativity of *β*-HCG values. Pathological examination confirmed that an omental ectopic pregnancy and chorionic villi were found between the blood clot and the adipose tissue. Nowadays she is pregnant.

## 3. Discussion

Abdominal pregnancy represents 1,4% of cases, associated with a mortality rate seven times higher than nonabdominal cases [2]. Usually an abdominal pregnancy is a secondary implantation of a tubal pregnancy; conversely a primary abdominal pregnancy implants directly in the abdominal cavity and its organs, except for tubes and ovaries. For the diagnosis of primary omental pregnancy, Studdiford's criteria need to be fulfilled (normal tubes and ovaries, absence of uteroperitoneal fistula, presence of a pregnancy related solely to the peritoneal surface without signs of tubal pregnancy first) [3]. According to Studdiford's criteria and with the histological evidence of the growth of trophoblast in the omental tissue, our case represents a rare case of primary omental pregnancy in a fertile woman (11 cases have been described in the literature from 2004) [2, 4–12]. Diagnosis of omental pregnancy is not simple, clinical signs are not specific, vaginal ultrasound is not always accurate, and laparoscopy approach is limited by a narrow visual field and lack of tactile sensation [3]. However, some authors reported successes with laparoscopic approach [7, 9–12]; most of the literature's cases are treated with laparotomy [4] after the rupture of the sac. In our case, clinical signs and vaginal ultrasound deposed for a preoperative diagnosis of ruptured tubal pregnancy but laparoscopic exploration found normal adnexa. The growth of *β*-HCG value was interpreted as a sign of PUL (pregnancy of unknown location); so, according to the literature data, a treatment with methotrexate was started, without success [13, 14]. In view of the hemodynamic instability, a laparotomy was done and the omental pregnancy was found. In conclusion, omental pregnancy is difficult to identify and clinicians should always consider the possibility of an abdominal pregnancy when pelvic organs are normal in a patient with PUL, abdominal pain, and hemoperitoneum.
